# Electrochemically Triggered Supramolecular Polymerization Under Kinetic Control

**DOI:** 10.1002/anie.8733637

**Published:** 2026-06-24

**Authors:** Eun Gyu Lee, Jeongse Yun, Hyoung Wook Kang, Daeun Jung, Seung‐Ryong Kwon, Jong Hwa Jung, Sung Ho Jung

**Affiliations:** ^1^ Department of Chemistry Gyeongsang National University Jinju Republic of Korea; ^2^ Research Institute of Molecular Alchemy Republic of Korea Department Gyeongsang National University Jinju South Korea; ^3^ Research Institute of Advanced Chemistry Republic of Korea Department Gyeongsang National University Jinju South Korea

**Keywords:** kinetic control, pathway complexity, perylene diimide, redox, supramolecular polymerization

## Abstract

Stimuli‐responsive supramolecular polymerization with high precision is essential for developing adaptive materials with programmable kinetics and functions. Here, we present a redox‐responsive strategy that integrates chemical redox reactions and electrochemical potential to direct the self‐assembly of a perylene diimide–histidine (**PDI**–**His**). A chemical redox process with sodium dithionite (SDT) rapidly converts kinetically trapped dimeric aggregates (Agg‐I) of **PDI**–**His** stabilized by intramolecular hydrogen bonding into thermodynamically favored helical nanofibers (Agg‐II), enabling the preparation of seeds with tunable lengths. Electrochemical potential application also induces reorganization of Agg‐I into Agg‐II, accompanied by morphological evolution, and improved conductivity via enhanced π–π stacking. Importantly, the redox‐cycle‐driven supramolecular reorganization was achieved not only on electrode surfaces through electrochemical stimuli but also through seeded‐living supramolecular polymerization using seeds generated via both chemical and electrochemical kinetic pathways, yielding nanofibers with predictable lengths. This combined chemical‐ and electrochemical‐redox approach provides an adaptable platform for controlling pathways in supramolecular polymerization, advancing the design of stimuli‐responsive materials for applications in electronics, sensing, catalysis, and bioinspired systems.

## Graphical abstract

Chemical and electrochemical stimuli enable controlled supramolecular polymerization of perylene diimide–histidine (**PDI**–**His**). Redox processes mediate the transformation of kinetically trapped aggregates into thermodynamically stable nanofibers, thereby supporting seeded growth. This approach provides a strategy for regulating self‐assembly pathways and the electrochemical properties of supramolecular materials.

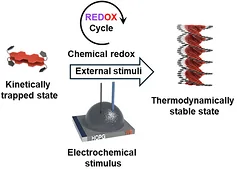

## 1 Introduction

Stimuli‐responsive supramolecular materials are crucial in the design of advanced functional systems as they can reversibly modulate both structural and functional properties in response to external stimuli [1, 2, 3, 4]. Such adaptive materials provide unique opportunities to integrate controllable molecular organization with bioinspired functionality as many biological assemblies are regulated by reversible redox signals and energy‐driven processes. For example, in living systems, redox gradients and ATP/GTP hydrolysis precisely regulate protein assembly and disassembly with remarkable spatial and temporal control [5]. Inspired by natural strategies, bioinspired approaches not only enable fine control over supramolecular polymerization but also open avenues for developing adaptive materials for bioelectronics, catalysis, and sensing, where precise spatiotemporal control over assembly and electron transport is essential [6, 7, 8].

Supramolecular polymerization represents a dynamic process in which monomers self‐assemble into well‐defined polymeric architectures through non‐covalent interactions. This process is typically driven by reversible interactions, such as hydrogen bonding, π–π stacking, and van der Waals forces, which enable the formation of highly ordered and functional soft materials [9, 10, 11]. Kinetic control provides a route to access metastable states with unique properties that are inaccessible under thermodynamic control, thereby creating pathway complexity and enabling the design of non‐equilibrium states with tailored functionalities [12, 13, 14]. One of the most intriguing aspects of non‐equilibrium systems is their ability to respond to diverse external stimuli, including temperature, pH, light, and electrochemical inputs [15]. Such stimuli‐responsive characteristics allow supramolecular assemblies to undergo reversible structural transformations, adapting their morphology and functionality. Specifically, redox‐responsive self‐assembly processes emerged as a powerful approach in which external energy inputs, such as chemical fuels, drive the spontaneous formation and transformation of supramolecular architectures. Recently, numerous chemically fueled self‐assembly processes have been reported, exhibiting characteristics of non‐equilibrium states, including dynamic instability [16, 17, 18]. In particular, chemically fueled redox cycling using reductants such as sodium dithionite has been widely utilized to regulate assembly and disassembly processes in PDI‐based supramolecular systems under non‐equilibrium conditions. However, redox‐cycle‐driven supramolecular reorganization from kinetically trapped states into thermodynamically favored supramolecular polymers remains largely unexplored. These methods have enabled the development of dynamic supramolecular systems that can adapt to environmental changes, paving the way for smart materials with responsive functions.

Perylene diimides (PDIs) derivatives have gained prominence as versatile building blocks for constructing self‐assembled architectures owing to their outstanding photophysical properties, chemical stability, and robust π–π stacking interactions [19, 20]. These features contributed to PDI‐based supramolecular architectures that are highly suitable for various applications, including organic electronics, photovoltaics, and sensing technologies [21, 22, 23, 24]. Furthermore, the unique redox‐responsive characteristic incorporating PDI cores with high electron mobility has been extensively explored [25, 26, 27], which induced pathways in PDI‐based systems expand their functional scope by enabling reversible transformation in supramolecular architecture [28, 29, 30]. Although redox‐responsive amino acid‐functionalized PDI assemblies have previously been investigated mainly in supramolecular hydrogel systems [30], electrochemically controlled pathway switching from kinetically trapped states into helical supramolecular polymers remains largely unexplored. Through precise electrochemical modulation, it is possible to induce adaptive responses and functional switching in such assemblies. The inherent redox properties of the PDI core play a central role in facilitating electron transfer processes that underpin the modulation of assembly dynamics, enabling the development of materials with novel electronic and optical properties.

Herein, we report a redox‐responsive strategy that combines chemical and electrochemical redox control to enable pathway‐dependent hierarchical self‐assembly of the **PDI**–**His** derivative under kinetic control. This system undergoes supramolecular reorganization from a kinetically trapped state to a thermodynamically favored state upon exposure to chemical reducing agents or electrochemical potentials as triggers. Importantly, the redox cycle induces structural reorganization of kinetically trapped states into supramolecular seeds that subsequently facilitate seeded supramolecular polymerization. By systematically investigating these dual‐responsive pathways, we establish a methodology for kinetic control in redox‐responsive supramolecular polymerization. Our approach is expected to provide a general platform for developing smart and bioinspired materials with tailored structural, electronic, and functional properties for applications in electronics, catalysis, sensing, and beyond.

## 2 Results and Discussion

### 2.1 Molecular Design for the Redox‐Responsive Supramolecular Polymerization

**SCHEME 1:**
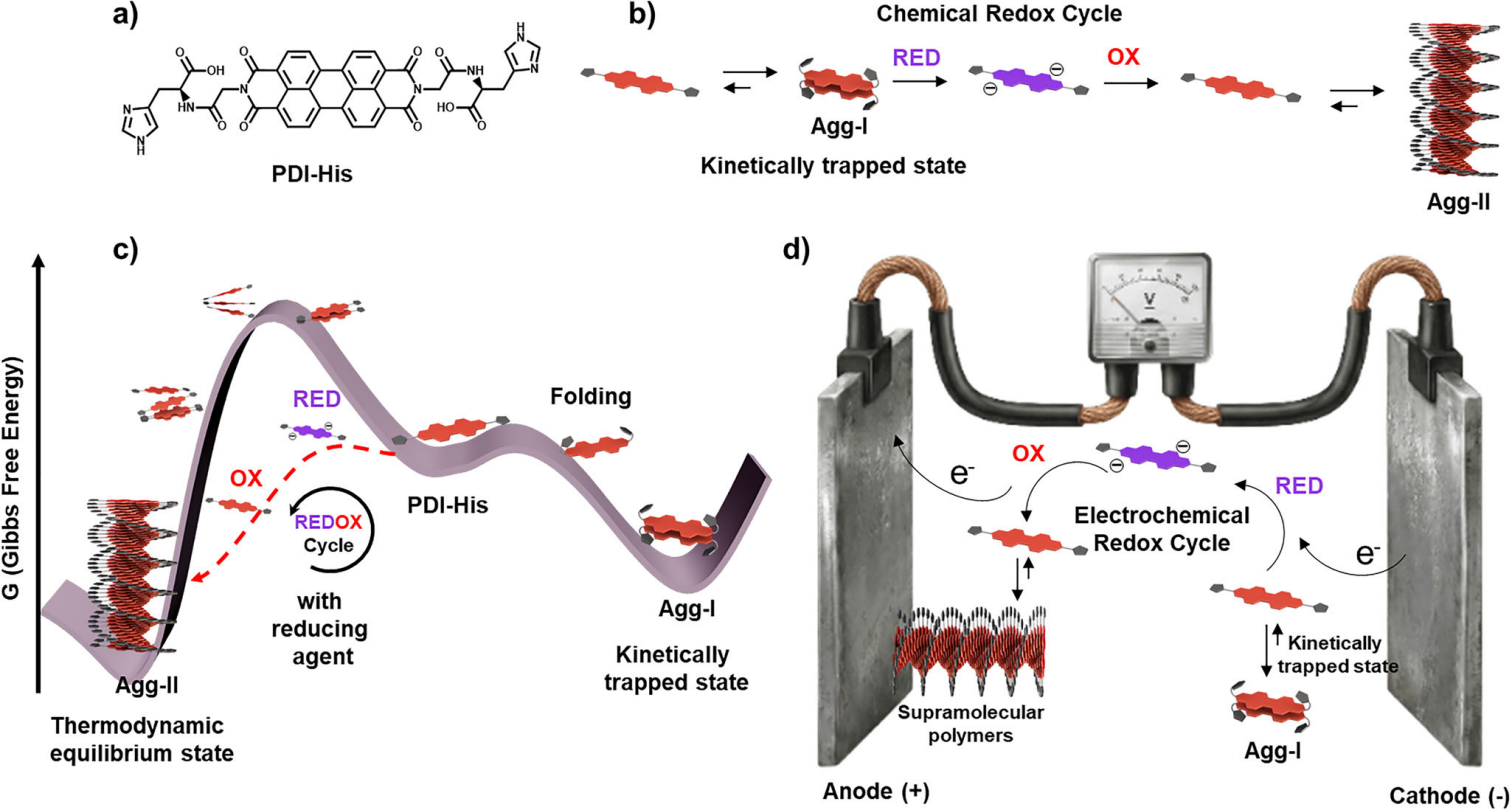
(a) Molecular structure of **PDI‐His**. (b) Schematic illustration of the chemical redox process of **PDI‐His** assemblies in the presence of SDT (sodium dithionite), (c) Gibbs free energy landscape of **PDI**–**His** assemblies with and without reducing agent (SDT), and (d) Electrochemical responsive redox process of **PDI**–**His** assemblies.

Herein, we designed a redox‐responsive supramolecular system in which a perylene diimide (PDI) core is covalently functionalized with an *L*‐histidine (His) unit via a glycine (Gly) linker. **PDI**–**His** was synthesized by condensing commercially available perylene‐3,4:9,10‐tetracarboxylicbis‐anhydride with an equimolar amount of Gly–His in the presence of imidazole, affording the product as a red solid in 51% yield (Scheme S1). Detailed synthetic procedures and characterization data are provided in the Supporting Information. In molecular design, the glycine‐linked His moiety plays a critical role in regulating the supramolecular assembly behavior through conformational flexibility and directional intra‐ and intermolecular hydrogen‐bonding interactions, which are key factors in fine‐tuning the generation of kinetically trapped species. In particular, the Gly–His moiety engages in strong intramolecular hydrogen bonding, stabilizing a kinetically trapped dimeric formation (Agg‐I). These dimers further self‐assemble into one‐dimensional helical supramolecular polymers with inherent chirality. Based on the redox properties of the PDI core, both chemical and electrochemical stimuli can induce conformational reorganization. In the folded dimeric state, Agg‐I represents a kinetically trapped intermediate. Upon chemical or electrochemical redox triggers, the supramolecular system undergoes kinetically controlled growth, transforming into thermodynamically favored aggregates (Agg‐II) (Scheme 1).

### 2.2 Self‐Assembly Behavior

Initially, we investigated the self‐assembly characteristics of **PDI–His** in a dimethylsulfoxide (DMSO) and water (H_2_O) solvent mixture. A solvent composition‐dependent study using UV–vis spectroscopy revealed the self‐assembling behavior of **PDI‐His**. In pure DMSO, the typical absorption bands of molecularly dissolved **PDI–His** (100 µM) with vibronic features were observed with the shoulder at 460 nm and two well‐resolved peaks at 490 and 527 nm, respectively, corresponding to the A_0‐2_, A_0‐1_, and A_0‐0_ transitions due to π–π* transitions of the PDI chromophore. Increasing the water content in the DMSO/water mixture from 0% to 60% results in a clear hypsochromic shift in the absorption spectrum, with a maximum absorption band observed at 496 nm. At a water fraction of 60%, a noticeable loss of vibronic fine structure is observed, accompanied by precipitation. Consequently, the vibronic signal pattern of monomeric **PDI–His**, corresponding to A_0‐1_ and A_0‐0_, is inverted in the DMSO/water mixture (5:5 v/v) (Figure S1). The A_0‐1_/A_0‐0_ ratio increases with increasing water fraction, suggesting tendencies toward dimerization. This finding aligns with previous studies that highlight similar behavior in related systems [31, 32]. This aspect will be discussed in more detail later.

**FIGURE 1:**
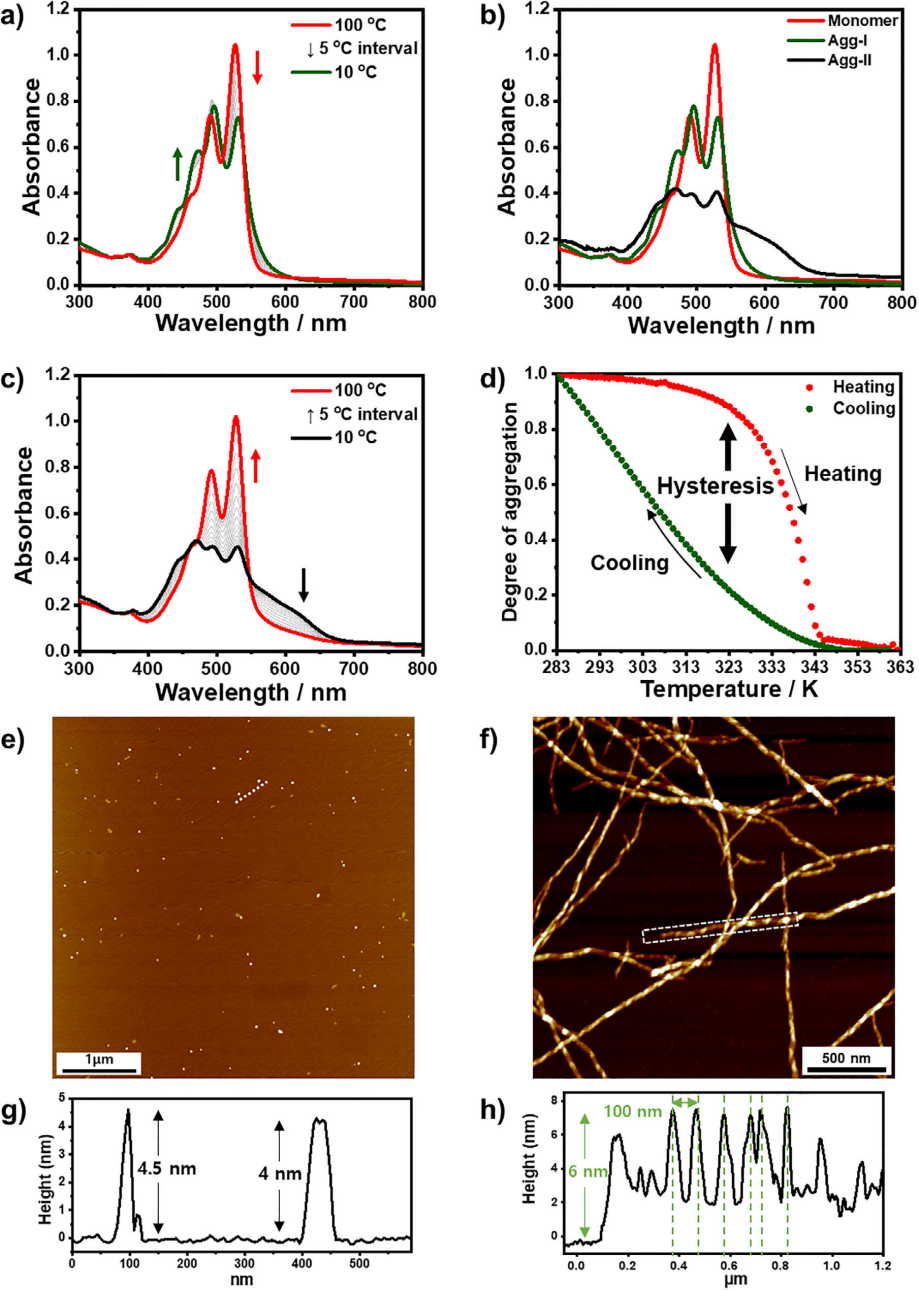
(a) Temperature‐dependent UV–vis spectra changes of Agg‐I. (b) UV–vis spectra of monomeric **PDI‐His** in DMSO, Agg‐I, and Agg‐II. (c) Temperature‐dependent UV–vis spectra changes of Agg‐II. (d) Temperature‐dependent degree of aggregation (*α_agg_*) of **PDI–His** observed in cooling (Agg‐I, green) and heating (Agg‐II, red) processes at a rate of 1K min^−1^. All UV–Vis spectra of **PDI**–**His** were recorded at a concentration of 100 µM in a mixed DMSO and H_2_O (5:5 v/v). AFM images of self‐assembled (e) Agg‐I and (f) Agg‐II. Height profiles of (g) Agg‐I and (h) Agg‐II corresponding to the white dashed lines and area in panels (e) and (f).

To further explore self‐assembling behavior, we conducted a study on the supramolecular polymerization of **PDI–His** in a solvent mixture of DMSO and H_2_O (1:1 v/v) by examining temperature‐dependent changes in absorption spectra (Figure 1a). Upon cooling to 283 K, the absorption maximum of **PDI–His** undergoes a hypsochromic shift to 496 nm, accompanied by a decrease in absorption intensity (hypochromic effect). These spectral changes are indicative of enhanced intermolecular π–π interactions and aggregation of **PDI–His** (hereafter referred to as Agg‐I). Additionally, the cooling curve of Agg‐I exhibited a sigmoidal transition, which could be fitted by using the dimerization model [33, 34]. The self‐assembling feature of Agg‐I was visualized by atomic force microscopy (AFM). In AFM observation, spin‐coating a solution of **PDI–His** (100 µM) in the DMSO/water mixture (1:1 v/v) onto a surface of highly oriented pyrolytic graphite (HOPG) revealed discrete nanoparticle‐like aggregates with a height of approximately 4 nm (Figure 1e,g).

Interestingly, after several days at room temperature, the absorption spectrum exhibited a new bathochromically shifted band at ∼600 nm, assigned to Agg‐II (Figure 1b). In contrast to the Agg‐I, the Agg‐II shows a broad absorption spectrum with a significant exciton bandwidth from approximately 400 nm to beyond 650 nm. The most striking spectral feature of Agg‐II is the bathochromically shifted absorption band at 600 nm, a characteristic commonly associated with the rotational displacement of the PDI core and a small rotational offset due to the slipped packing arrangement of PDIs [35, 36]. In parallel, circular dichroism (CD) spectra of Agg‐II exhibited a negative CD signal at 500 nm, corresponding to the main absorption band of the PDI chromophore (Figure S2). This enhanced chiroptical signal arises from exciton coupling among π–π stacked PDI units organized into a left‐handed helical arrangement, which originates from the *L*‐histidine moiety in **PDI–His**. To further investigate the origin of this supramolecular helicity, the geometry optimization using density functional theory (DFT) calculations at B3LYP/6‐31G(d) level was performed on a trimeric **PDI–His** assemblies. The optimized structure revealed slipped π‐π stacking together with intermolecular amide C═O···H─N hydrogen‐bonding interactions and a small average tilting angle (∼3.2°) between adjacent PDI planes, indicating a slightly tilted and rotationally displaced packing geometry (Figure S3). We propose that this asymmetric slipped‐stacking arrangement originates from the *L*‐histidine side chains and contributes to the formation of left‐handed helical nanofibers. Consistently, AFM observations revealed left‐handed helical nanofibers with an average height of ∼6 nm and a helical pitch of ∼100 nm (Figure 1f). X‐ray diffraction (XRD) analysis provided further insights into the packing structures of Agg‐I and Agg‐II. Agg‐I exhibited a broad diffraction feature corresponding to a d‐spacing of approximately 3.37 Å, whereas Agg‐II showed a π–π stacking distance of approximately 3.56 Å. Although Agg‐I exhibits a shorter intermolecular distance, its broad diffraction pattern indicates a relatively disordered molecular packing within nanoparticles. In contrast, Agg‐II exhibits a more ordered supramolecular organization associated with helical nanofibers (Figure S4) [37]. However, due to the broad resonances and precipitation observed at highly concentrated solutions of **PDI–His** in the mixture of DMSO‐D_6_/D_2_O (1/1 v/v), it hinders the use of NMR experiments to elucidate the packing information of both Agg‐I and Agg‐II. As a result, these observations imply that kinetically controlled Agg‐I as nanoparticles transformed into thermodynamically favored states as helical nanofibers (Agg‐II). Notably, after complete transformation of **PDI–His** from Agg‐I to Agg‐II at room temperature, the resulting Agg‐II exhibited a non‐sigmoidal transition upon heating, indicating a cooperative mechanism. The temperature‐dependent degree of aggregation for Agg‐I and Agg‐II was determined from the apparent absorption coefficients at 527 and 600 nm, respectively. This behavior resulted in a clear thermal hysteresis between the cooling (Agg I) and heating (Agg II) processes (Figure 1d), further supporting the distinct kinetic and thermodynamic nature of the two aggregated states.

### 2.3 Revealing Pathway Complexity With Thermodynamic Parameters

**FIGURE 2:**
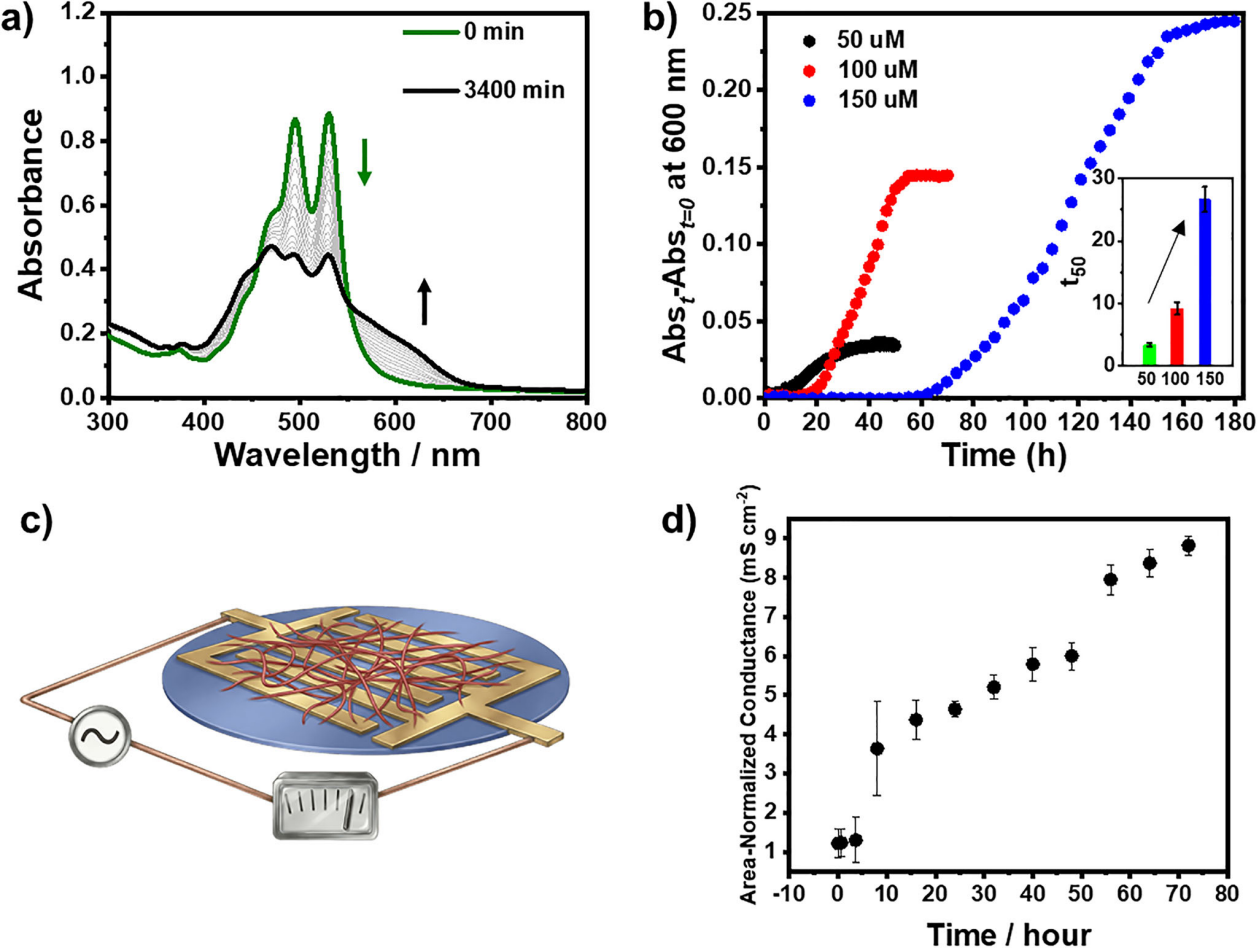
(a) Time‐dependent absorption spectral changes of **PDI–His** (100 µM) observed after fast cooling from 373 to 298 K in a mixture of DMSO and H_2_O (5:5 v/v). (b) Plot of absorption at the given wavelength as a function of time at different concentrations. The insert in (b): dependence of **PDI–His** concentration on the lag time for transformation from Agg‐I into Agg‐II under stirring at 400 rpm. The lag time was defined as the time at which 50% of the transformation is complete. (c) Schematic illustration of the electrochemical transport measurement setup in which the electrical conductivity of **PDI–His** was measured. (d) Time‐dependent conductivity of the transformation from Agg‐I into Agg‐II.

To gain a deeper insight into the dynamic self‐assembling behavior of **PDI–His**, we conducted a kinetic study on the transformation between Agg‐I and Agg‐II under stirring at 400 rpm. The time‐dependent change of Agg‐I in absorption spectra at 298 K revealed that kinetically formed Agg‐I transforms into Agg‐II within a period of about 60 h (Figure 2a,b). The plot of absorption at 600 nm shows a gradual increase, indicating the transformation of Agg‐I into Agg‐II. This nonlinear sigmoidal profile is characteristic of the autocatalytic process in accordance with the cooperative mechanism underlying the formation of Agg‐II [38, 39]. In the kinetic profile, the lag time (defined as the time at which the transformation is 50% completed) increased with the concentration of Agg‐I. This observation clearly indicates that Agg‐I acts as an off‐pathway intermediate in the formation of Agg‐II, thereby contributing to the inhibition of the spontaneous nucleation process for Agg‐II. Thus, monomeric PDI‐His could directly form Agg‐II by following a nucleation‐elongation growth mechanism. Given the expected dynamic transformation of the π‐conjugated system, we next investigated the effect of supramolecular transformation on charge transport using electrochemical impedance spectroscopy (EIS). The impedance measurements were performed under static (no stirring) conditions to maintain stable electrical contact between the electrodes. During the transformation from Agg‐I to Agg‐II (100 µM), a significant increase in electrical conductivity was observed after approximately 8 h (Figure 2c,d), correlating with the morphological evolution from nanoparticles (Agg‐I) to elongated and ordered helical nanofibers (Agg‐II). Notably, the conductivity enhancement was detected prior to complete spectroscopic conversion into Agg‐II. This difference likely arises because EIS is highly sensitive to the formation of electrically connected supramolecular pathways between the electrodes, whereas UV–vis spectroscopy reflects ensemble‐averaged aggregation behavior throughout the bulk solution. This enhanced conductivity is attributed to the reorganization of the **PDI–His**, where the alignment of π‐conjugated systems within the helical nanofibers becomes more ordered, thus improving the pathways for electron mobility [40].

To understand the kinetically trapped dimeric Agg‐I formed as an off‐pathway intermediate during the time evolution process, we further investigated the hydrogen‐bonding interactions by Fourier‐transform infrared (FT‐IR) spectroscopy. The FT‐IR spectrum of Agg‐I exhibited a C═O stretching band at approximately 1644 cm^−1^ in a DMSO/water mixture, compared with the corresponding band of monomeric **PDI–His** at 1649 cm^−1^ in pure DMSO (Figure S5). In addition, a characteristic band associated with intramolecular amide‐imidazole hydrogen‐bonding interactions was observed at 1311 cm^−1^ [41, 42]. These results suggest that **PDI–His** assumes a folded conformation stabilized by intramolecular hydrogen bonding, which promotes dimerization in aqueous media via hydrophobic interactions [43]. This folded dimeric formation likely inhibits the propagation of **PDI–His** into extended supramolecular assemblies, thereby preventing the formation of helical nanofibers (Agg II) under kinetic conditions. In contrast, the C═O stretching band of Agg‐II was further shifted to 1638 cm^−1^, indicating hydrogen‐bonding interactions of amide group rearranged during the transformation (Figure S5) [44]. To gain further structural insight, we then explored geometry optimization using density functional theory (DFT) calculations at B3LYP/6‐31G(d) level. The optimized structures of the monomeric **PDI–His** showed two key intramolecular hydrogen bonds: one between the amide hydrogen and imidazole (d(N─H···N─C) = 2.02 Å), and another between the hydroxyl group and carbonyl group (d(O─H···O═C) = 1.75 Å) (Figure S5). In addition, DFT calculations on a dimer of two **PDI─His** showed compact intermolecular packing stabilized by π–π interactions between the PDI cores, together with intramolecular hydrogen bonding interactions involving the histidine moieties (Figure S6). These interactions corroborate the intramolecular hydrogen bonding observed in the FT‐IR spectra and support the presence of compact dimeric structures in Agg‐I.

To elucidate the dynamic self‐assembling behavior of **PDI–His** in terms of its thermodynamic properties, concentration‐ and temperature‐dependent UV–vis experiments were performed (Figures S7–S10). During the cooling process, the temperature‐dependent absorption spectra of **PDI–His** revealed a sigmoidal transition at 527 nm. The degree of Agg‐I as a function of the total concentration of **PDI–His** obtained by fitting the apparent absorption coefficients at 527 nm to the monomer‐dimer model [33, 34]. The fitting to the monomer‐dimer model yielded *K*_d_ at different temperatures (Table S1). The van't Hoff plot shows a linear relationship (correlation coefficient of 0.996), allowing for the determination of the standard enthalpy (Δ*H*°), entropy (Δ*S*°), and Gibbs free energy (Δ*G*°) at 298 K associated with the formation of Agg‐I, which were found to be of −98 ± 4.8 kJ mol^−1^, −241 ± 15.5 J mol^−1^K^−1^, −26 ± 6.7 kJ mol^−1^, respectively (Figure S8). The disassembly process of the thermodynamic state of Agg‐II was monitored using temperature‐dependent UV−vis spectroscopy. As the temperature increased from 298 to 325 K, the characteristic lowest energy absorption band at *λ* = 600 nm disappeared, and the broad absorption spectrum of Agg‐II converted into monomeric **PDI–His**, exhibiting a non‐sigmoidal transition with an elongation temperature (*T*_e_) of 343 K at a concentration of 100 µM. The non‐sigmoidal transition could be properly fitted using a cooperative model [45]. Upon diluting the total concentration of PDI‐His (*C*_t_), the *T*_e_ decreased with a linear relationship in the van't Hoff plot, from which the standard enthalpy (Δ*H*°), entropy (Δ*S*°), and Gibbs free energy (Δ*G*°) at 298 K were estimated to be of −109 ± 1.1 kJ mol^−1^, −240 ± 3.0 J mol^−1^K^−1^, −38 ± 0.3 kJ mol^−1^, respectively (Figure S10 and Table S2). The obtained Δ*H*° and Δ*S*° values are comparable to those reported for structurally related PDI‐based cooperative supramolecular polymerization systems [46, 47, 48]. Although the temperature‐dependent degree of aggregation was monitored during the heating process, the thermodynamic parameters extracted from the van't Hoff analysis describe the cooperative supramolecular polymerization. Therefore, the negative entropy value reflects the increased structural ordering and restricted molecular motion associated with supramolecular polymerization [11]. The observation of a distinct elongation temperature (*T*_e_) together with a nonlinear sigmoidal kinetic profile is characteristic of cooperative supramolecular polymerization involving a nucleation‐elongation mechanism. These analytical results support the formation of helical fibrous Agg‐II through a cooperative nucleation‐growth process [11]. Namely, dimeric Agg‐I was kinetically trapped as a metastable aggregate, which is an off‐pathway intermediate with regard to the thermodynamically stable Agg‐II. As a result, the spontaneous nucleation for Agg‐II was inhibited, while this kinetic trapping is attributed to the formation of intramolecular hydrogen bonds within the folded formation of **PDI–His**. These thermodynamic parameters clearly demonstrate the dynamic self‐assembling process over time, emphasizing that the kinetically trapped state of Agg‐I eventually transforms into the thermodynamically favored Agg‐II.

### 2.4 Living Supramolecular Polymerization by Redox Processes

Inspired by the above findings and the work of George and co‐workers [49], we investigated redox‐responsive supramolecular polymerization. The kinetically trapped state Agg‐I presents a promising intermediate for modulating self‐assembly pathways via external stimuli. By exploiting the redox cycle of the PDI core using a reduction agent [17, 50, 51], we aimed to regulate the dynamic transformation from Agg‐I to thermodynamically favored Agg‐II.

**FIGURE 3:**
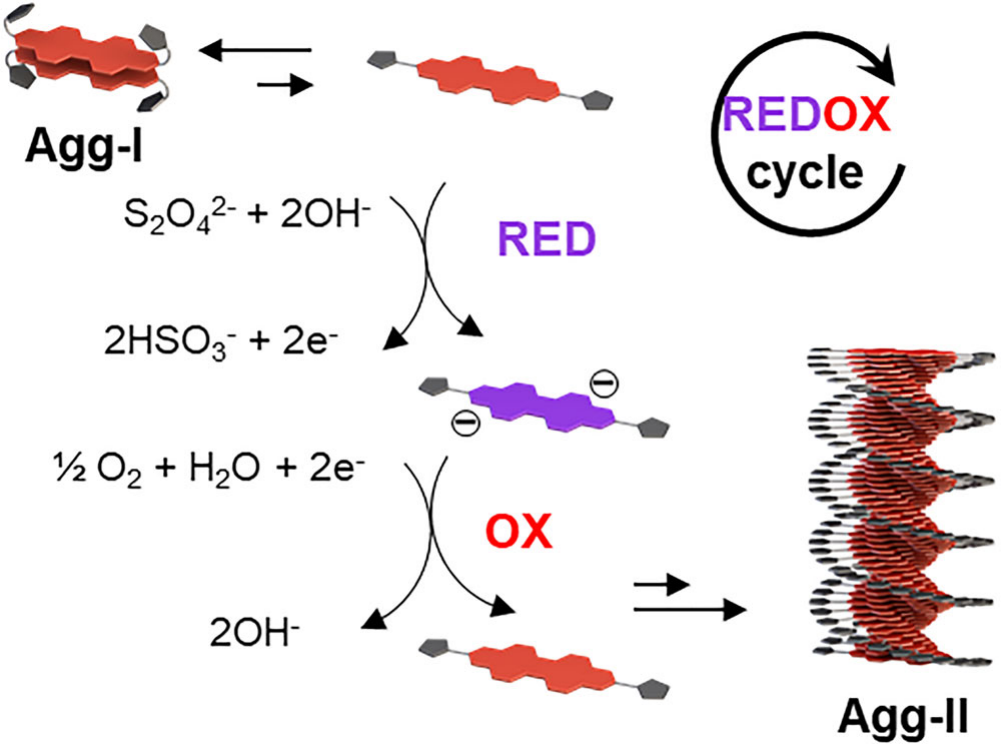
Schematic representation of the redox cycle of **PDI–His**: Upon addition of Na_2_S_2_O_4_ (SDT), **PDI–His** is reduced to its dianionic form (PDI^2^^−^). Subsequent spontaneous oxidation in air first generates the radical anion (PDI•^−^), which then converts to the neutral **PDI–His**.

**FIGURE 4:**
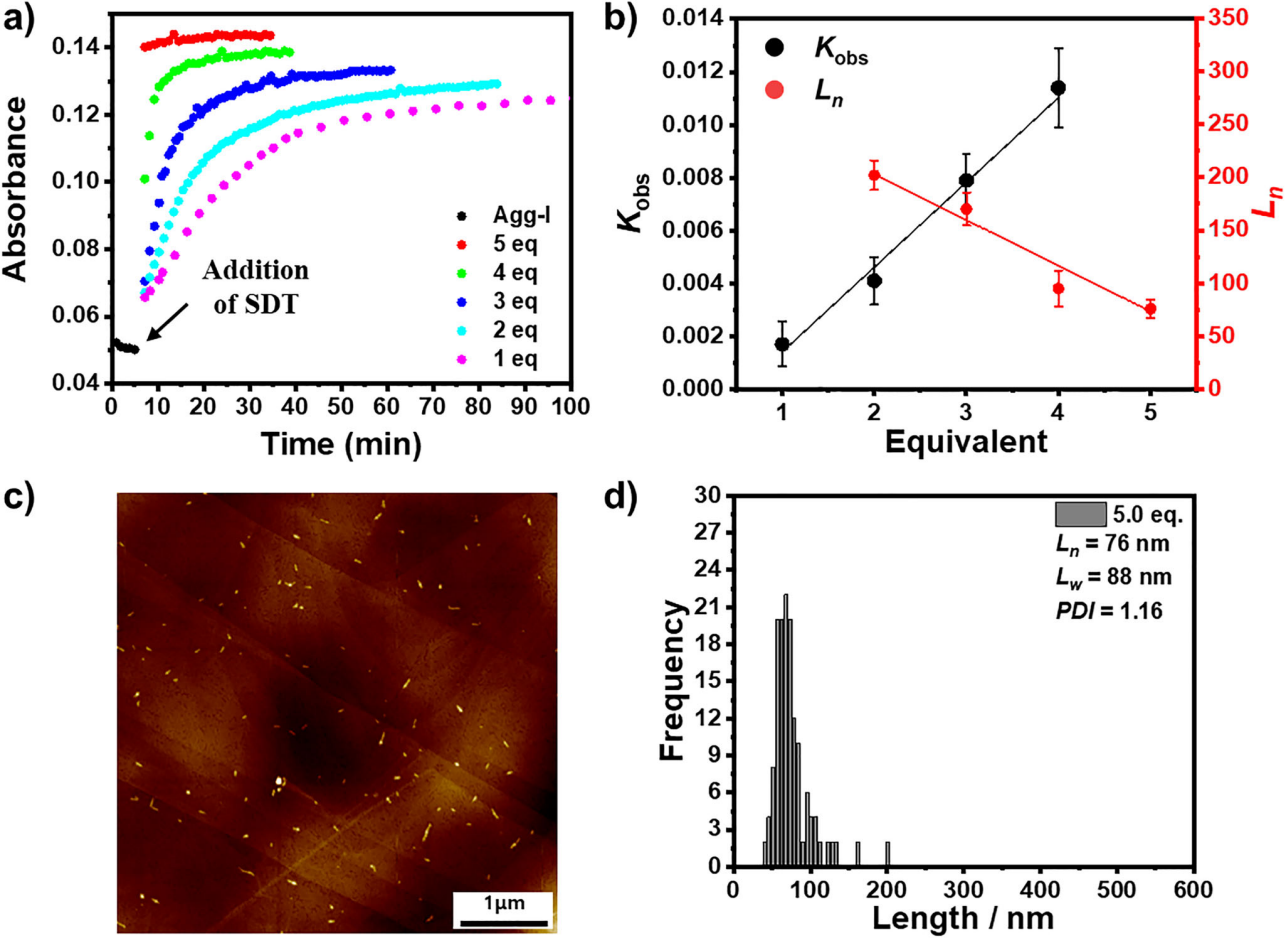
(a) Kinetic profile showing the transformation of Agg‐I (100 µM) into Agg‐II upon the addition of SDT (1.0–5.0 eq.) monitored by absorption changes at 600 nm. (b) Plot of observed initial rate (*K*_obs_) forming Agg‐II and number‐average length (*L*_n_) of Agg‐II as a function of SDT concentrations (2.0–5.0 eq.). (c) AFM image and corresponding (d) length distribution of Agg‐II obtained after addition of 5.0 eq. of SDT to Agg‐I.

To probe the redox‐responsive behavior of **PDI–His**, we performed chemical reduction experiments by adding sodium dithionite (SDT) to the kinetically trapped state of Agg‐I. The chemical redox process of **PDI–His** (100 µM) with sodium dithionite (SDT) in a mixture of DMSO/water (1:1 v/v) was monitored for its characteristic spectral features, which are indicative of redox states in supramolecular polymerization (Figure 3). Upon addition of SDT (5.0 eq) to Agg‐I, the absorption spectrum changed immediately, resembling that of Agg‐II. Notably, the transformation into Agg‐II occurred without a lag phase, suggesting that the redox process significantly accelerates the nucleation step for Agg‐II formation. Systematic variation of SDT equivalents (1.0–5.0) revealed a clear increase in absorbance at 600 nm, with the most pronounced effect observed at 5.0 eq. of SDT. The kinetic profiles showed that the initial rate of Agg‐II formation increased proportionally with SDT concentration, demonstrating that the redox‐driven assembly proceeds as a function of the reducing agent level (Figure 4 and S11). These results highlight the ability of SDT to modulate the polymerization kinetics, thereby enabling precise control over the self‐assembly process. This acceleration is probably attributed to the generation of transient reduced states of the PDI core (namely, the radical anion (PDI^•^^−^) and dianion (PDI^2^^−^)) upon SDT treatment. Under ambient conditions, these reduced species rapidly revert to their neutral state due to the presence of atmospheric oxygen. Interestingly, the redox stability of Agg‐II was maintained even upon exposure to excess SDT (up to 5 eq.), as no significant changes were observed in its absorption spectrum with morphology (Figure S12). This result indicates that the higher thermodynamic stability of Agg‐II, relative to Agg‐I, enables it to retain its structural stability during redox cycling. In contrast, at higher SDT equivalents (40 eq.), the reduced species persist longer, as evidenced by absorption spectra with distinct color changes (Figure S13) [28]. Under deoxygenated conditions using a sealed cuvette, the reduced PDI species were rapidly generated upon addition of SDT and remained stable over time without undergoing the characteristic spectral transition associated with Agg‐II formation (Figure S14). After 150 min, the absorption maximum of the reduced PDI species (**PDI–His** ^2−^) exhibited a redshift from 496 to 546 nm in the presence of excess SDT (5.0 eq.) (Figure S14), indicating stabilization of the reduced state under deoxygenated conditions. These observations suggest that transient reduction alone is insufficient to induce transformation into Agg‐II and that subsequent reoxidation of the reduced PDI species by atmospheric oxygen plays a critical role in promoting supramolecular reorganization into the thermodynamically favored helical nanofiber state. To further probe the influence of reduction on the molecular conformation of **PDI–His**, DFT geometry optimization was additionally performed for the dianionic species (**PDI–His^2−^**). Compared with neutral **PDI–His**, the optimized dianionic structure showed an increase in the intramolecular amide N─H···N(imidazole) interaction from 2.02 Å to approximately 2.53 Å, indicating weakening of the folded conformation stabilized by intramolecular hydrogen bonding (Figure S15). The dianionic structure also adopted a more open conformation, suggesting that electrostatic repulsion generated upon reduction of the PDI core may facilitate outward reorientation of the histidine moieties and promote π–π stacking during transformation into Agg‐II.

Interestingly, supramolecular polymers of Agg‐II were successfully promoted via redox processes. AFM analysis revealed that the resulting helical nanofibers exhibited a shorter length distribution, with a number‐averaged length (*L*_n_) of 76 nm and a polydispersity index (PDI, *L*_w_/*L*_n_) of 1.16 (Figures 4 and S16). This is probably due to more frequent nucleation events during the redox‐induced assembly. At 5.0 equivalents of SDT, a larger number of smaller nanofibers was observed compared to those formed at lower SDT concentrations. Dynamic light scattering (DLS) measurements supported these findings, showing a decreasing trend in the average hydrodynamic diameter with increasing SDT concentration (Figure S17), consistent with AFM observations (Figure S18). These results suggest that redox‐mediated access to Agg‐II from kinetically trapped Agg‐I provide a more effective strategy for seed preparation than conventional sonication. In particular, the redox responsiveness of the **PDI–His** system allows precise control over the polymerization process, enabling modulation of fiber length and structural characteristics through the concentration of the reducing agent (Figure 4b). Unlike sonication, which typically yields seeds of uniform length, the redox‐based approach affords seeds with either uniform or varied lengths depending on the SDT concentration, thereby offering flexibility in customizing supramolecular polymers.

**FIGURE 5:**
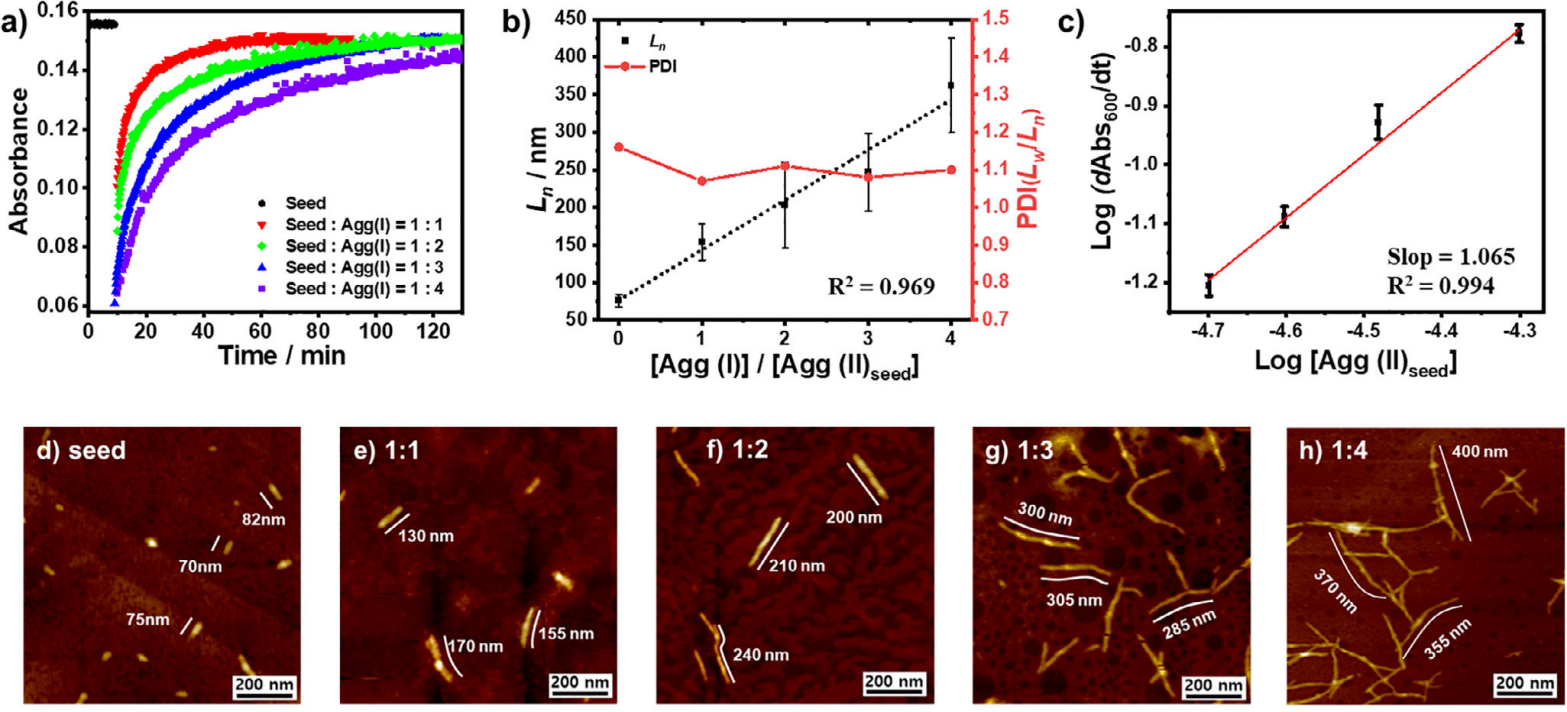
(a) Time‐dependent absorption changes at 600 nm showing seeded supramolecular polymerization upon addition of Agg‐I into the Agg‐II_seed_ under conditions ([Agg‐II_seed_]: [Agg(I)] = 1:1, 1:2, 1:3, 1:4, and stirring rate = 400 rpm). (b) Plots of the number‐averaged length (*L*_n_), weight‐averaged length (*L*_w_), and PDI (*L*_w_/*L*_n_) of the seed and supramolecular polymers as a function of the ratio of the total amount of Agg‐I to the initial amount of Agg‐II_seed_. (c) Log‐Log plot of the initial growth rate at 600 nm (s^−1^) as a function of Agg‐II_seed_ concentration, showing a linear relationship with a slope of 1.06. (d–h) AFM images obtained from the seeded supramolecular polymerization in a mixture of DMSO and H_2_O (5:5 v/v): at different ratios of [Agg(II)Seed]: [Agg(I)]; d) Agg‐II_Seed_, (e) 1:1, (f) 1:2, (g) 1:3, and (h) 1:4.

These findings prompted us to employ the small size of SPs as Agg‐II (denoted as Agg‐II_seed_) to seeded living polymerization. In this approach, we added different volumes of Agg‐II_seed_ solution to kinetically trapped nanoparticles (Agg‐I), with [Agg‐II_seed_]/[Agg‐I] volume ratios of 1:1, 1:2, 1:3, and 1:4. The polymerization process was initiated without a lag time (Figure 5a). The observed logarithm of the polymerization rate was linearly dependent on the logarithmic concentration of Agg‐II_seed_, indicating propagation processes driven by the redox‐prepared seed. AFM analysis of the size distribution of resulting Agg‐II further supported these observations (Figures 5 and S19). The linear relationship in the plot of fiber length versus [Agg‐IIseed]/[Agg‐I] ratios provides additional evidence for the living nature of the supramolecular polymerization. The number‐averaged lengths (*L*_n_) of the nanofibers were determined to be 154, 203, 247, and 362 nm for the [Agg‐IIseed]/[Agg‐I] volume ratios of 1:1, 1:2, 1:3, and 1:4, respectively. These results demonstrate that redox‐generated Agg‐II_seed_ acts as an effective nucleus for controlled supramolecular growth, enabling precise modulation of fiber length. This seeded approach provides a versatile platform for the bottom‐up fabrication of structurally defined materials with tunable hierarchical organization.

### 2.5 Electrode Stimulus‐Responsive Supramolecular Polymerization

To further expand the external control of supramolecular polymerization, we investigated the influence of heterogeneous electrochemical stimuli on the self‐assembly behavior of **PDI–His**. The redox properties of monomeric **PDI–His** were examined by cyclic voltammetry (CV) (Figure S20). The CV analysis revealed two well‐defined reduction waves at approximately −0.43 and −0.58 V (0.0 to −1.0V vs. Ag/AgCl), corresponding to the formation of the radical anion (PDI•^−^) and dianion (PDI^2^^−^), accompanied by a corresponding back‐oxidation process. The presence of two‐reduction processes in the monomeric state indicates pronounced redox activity, providing a basis for redox‐responsive modulation of the self‐assembly pathway. In contrast, the Agg‐II exhibited a relatively weak reduction wave at approximately −0.58 V (0.0V to −1.0V vs. Ag/AgCl) (Figure S20). The single reduction wave observed for Agg‐II suggests a more rigid and electronically stabilized packing arrangement, consistent with its thermodynamically favored aggregated state. These distinct electrochemical profiles indicate that the redox behavior of **PDI–His** is strongly dependent on its aggregated states, highlighting the role of kinetically trapped aggregates (Agg‐I) in governing electrochemical accessibility and reactivity.

**FIGURE 6:**
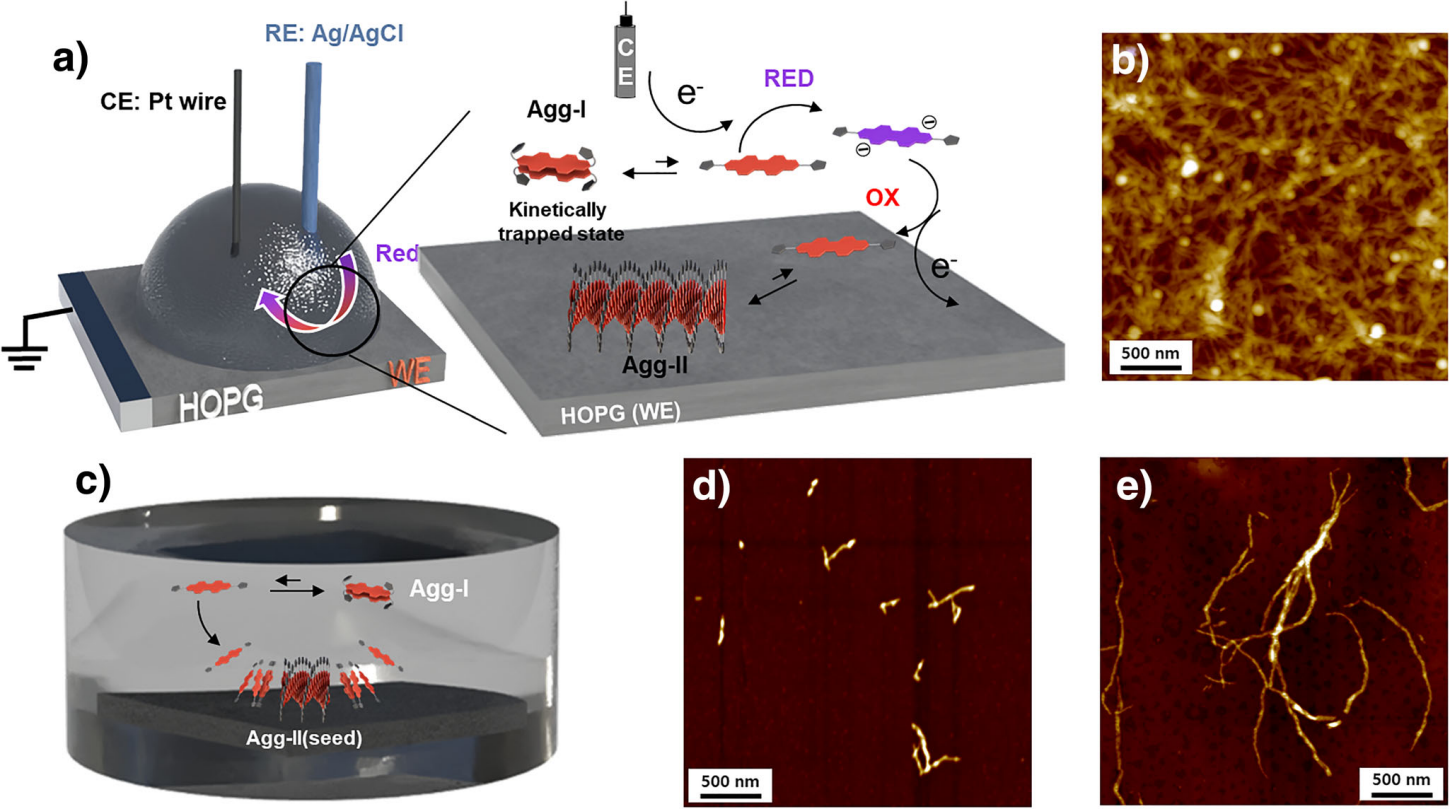
(a) Schematic illustration of electrode stimulus‐responsive **PDI–His** assemblies. (b) AFM image of Agg‐II prepared by electrochemical stimulation. (c) Schematic representation of seed‐initiated supramolecular polymerization achieved by immersing Agg‐II_seed_ on an HOPG substrate in a solution containing Agg‐I. (d) and (e) AFM image of the (e) elongated Agg‐II grown from (d) Agg‐II_seed_ on HOPG after seed‐initiated supramolecular polymerization.

To further investigate the influence of electrochemical input on supramolecular polymerization, an oxidative potential was applied to the solution containing Agg‐I. A highly oriented pyrolytic graphite (HOPG) electrode was employed as the working electrode, and an electrochemical potential of +1.0 V was subsequently applied under ambient conditions for 1 h. AFM revealed a distinct morphological transformation from isotropic nanoparticles (Agg‐I) to well‐defined helical nanofibers with microlength corresponding to Agg‐II (Figure 6a,b). This observation suggests that the application of oxidative potential is associated with redox processes occurring at the interface between the working electrode and the solution. In this context, reduced **PDI–His** species formed at the counter electrode under applied potential may undergo re‐oxidation at the HOPG working electrode‐solution interface under ambient conditions, which could promote nucleation and subsequent elongation processes. To further characterize the electrochemically generated Agg‐II species, CD and FT‐IR measurements were performed after collecting the assemblies from the electrochemically treated solution. The electrochemically generated Agg‐II exhibited CD and FT‐IR features comparable to those of solution of Agg‐II formed by time‐evolution, indicating similar chiral supramolecular organization and intermolecular hydrogen‐bonding interactions (Figures S21 and S22).

Although the length of helical nanofibers (Agg II) was not precisely controlled, the resulting helical nanofibers exhibited relatively short lengths by applying an electrochemical current for 10 min, with a number‐averaged length (*L*_n_) of 338 nm and a moderate dispersity (PDI = 1.22) (Figures 6d and S23). Such short Agg II nanofibers generated at the HOPG surface are therefore considered suitable candidates to act as seeds for supramolecular polymerization, even at the interface between the HOPG surface and the surrounding Agg I solution. To further assess this possibility, seeded growth experiments were performed using electrochemically generated seeds (Figure 6c). Agg II_seed_, prepared on the HOPG electrode by applying an electrochemical current for 10 min, was immersed in a solution containing Agg I. AFM observation revealed elongation of helical nanofibers to micrometer‐scale lengths from the termini of the electrochemically prepared seeds (Figure 6d,e), which is consistent with seed‐initiated supramolecular polymerization. Taken together, these results suggest that both chemical and electrochemical stimuli can generate seed species that can contribute to living supramolecular polymerization. The use of electrochemical stimuli to influence non‐covalent interactions and structural reorganization thus provides a viable strategy for externally regulating supramolecular polymerization, offering a promising approach toward electrochemically responsive materials with tunable properties.

## 3 Conclusion

We have demonstrated that both chemical redox and electrochemical stimuli can serve as effective external triggers to regulate the supramolecular polymerization of **PDI–His**, thereby providing a controllable platform for generating functional supramolecular materials with tunable structural and electronic properties. The kinetically trapped aggregates (Agg‐I), stabilized by intramolecular hydrogen bonding, represent a metastable state that can undergo redox‐mediated transformation into the thermodynamically favored helical nanofiber state (Agg‐II). This transformation is accompanied by enhanced charge delocalization, which is consistent with improved π–π stacking interactions in the assembled structures. Chemical reduction with sodium dithionite (SDT) promotes the transient formation of reduced PDI species (PDI^•−^ and PDI^2^^−^), which accelerates the nucleation process and suppresses the lag phase, leading to controlled seed formation with relatively narrow dispersity. Heterogeneous electrochemical stimuli further extend this level of control by inducing structural reorganization at the electrode–solution interface, thereby enabling the transformation of Agg‐I into Agg‐II. Importantly, the redox‐mediated transformation of kinetically trapped Agg‐I into thermodynamically stable Agg‐II provides an efficient strategy for supramolecular seed generation compared with conventional ultrasonication‐based methods. Furthermore, the redox‐responsive nature of the **PDI–His** system enables precise kinetic control over supramolecular polymerization, including tunable fiber growth and pathway‐dependent structural evolution through redox cycles. The combined influence of redox activity and supramolecular dynamics highlights how electron transfer processes can be employed to modulate self‐assembly pathways and electronic coupling in π‐conjugated systems. Collectively, these findings provide mechanistic insight into supramolecular polymerization under kinetic control and outline a versatile strategy for accessing non‐equilibrium, stimuli‐responsive supramolecular materials. The ability to regulate self‐assembly pathways and associated conductivity through complementary chemical and electrochemical inputs opens new opportunities for advanced applications in organic optoelectronics, chiroptical photonics, and adaptive supramolecular devices.

## Conflicts of Interest

The authors declare no conflicts of interest.

## Supporting information




**Supporting File**: The authors have cited additional references within the Supporting Information [52, 53, 54].

## Data Availability

The data that supports the findings of this study are available in the Supporting Information of this article.

## References

[1] X. Yan , F. Wang , B. Zheng , and F. Huang , “Stimuli‐Responsive Supramolecular Polymeric Materials,” Chemical Society Reviews 41 (2012): 6042–6065, 10.1039/c2cs35091b. 22618080

[2] X. Ma and H. Tian , “Stimuli‐Responsive Supramolecular Polymers in Aqueous Solution,” Accounts of Chemical Research 47 (2014): 1971–1981, 10.1021/ar500033n. 24669851

[3] J. Hoque , N. Sangaj , and S. Varghese , “Stimuli‐Responsive Supramolecular Hydrogels and Their Application in Regenerative Medicine,” Macromolecular Bioscience 19 (2019): 1800259, 10.1002/mabi.201800259. PMC6333493 30295012

[4] M. Crippa , C. Perego , and G. M. Pavan , “Non‐Trivial Stimuli‐Responsive Collective Behaviours Emerging From Microscopic Dynamic Complexity in Supramolecular Polymer Systems,” Nature Communications 16 (2025): 60150, 10.1038/s41467-025-60150-4. PMC12125348 40447580

[5] J. Deng and A. Walther , “ATP‐Driven Fueling and Tuning of Supramolecular Polymer Hydrogels,” Advanced Materials 32 (2020): 2002629, 10.1002/adma.202002629.

[6] A. Legrand , L. H. Liu , P. Royla , et al., “Spatiotemporal Control of Supramolecular Polymerization and Gelation of Metal–Organic Polyhedra,” Journal of the American Chemical Society 143 (2021): 3562–3570, 10.1021/jacs.1c00108. 33646776

[7] B. Mu , X. Hao , X. Luo , Z. Yang , H. Lu , and W. Tian , “Bioinspired Polymeric Supramolecular Columns as Efficient yet Controllable Artificial Light‐harvesting Platform,” Nature Communications 15 (2024): 1374, 10.1038/s41467-024-45252-9. PMC10827788 38291054

[8] S. Dhiman , A. Sarkar , and S. J. George , “Transient Dormant Monomer States for Supramolecular Polymers With Low Dispersity,” RSC Advances 8 (2018): 18913–18925, 10.1039/C8RA03225D. 35539685 PMC9080672

[9] H. Q. Peng , W. Zhu , W. J. Guo , et al., “Recent Advances in Supramolecular Polymers,” Progress in Polymer Science 136 (2023): 101635, 10.1016/j.progpolymsci.2022.101635.

[10] M. J. Webber , E. A. Appel , E. W. Meijer , and R. Langer , “Supramolecular Biomaterials,” Nature Materials 15 (2015): 13–26, 10.1038/nmat4474. 26681596

[11] T. F. A. De Greef , M. M. J. Smulders , M. Wolffs , A. P. H. J. Schenning , R. P. Sijbesma , and E. W. Meijer , “Supramolecular Polymerization,” Chemical Reviews 109 (2009): 5687–5754. 19769364 10.1021/cr900181u

[12] P. A. Korevaar , S. J. George , A. J. Markvoort , et al., “Pathway Complexity in Supramolecular Polymerization,” Nature 481 (2012): 492–496, 10.1038/nature10720. 22258506

[13] S. Ogi , T. Fukui , M. L. Jue , M. Takeuchi , and K. Sugiyasu , “Kinetic Control Over Pathway Complexity in Supramolecular Polymerization Through Modulating the Energy Landscape by Rational Molecular Design,” Angewandte Chemie International Edition 53 (2014): 14363–14367, 10.1002/anie.201407302. 25354399

[14] J. Matern , Y. Dorca , L. Sánchez , and G. Fernández , “Revisiting Complexity in Supramolecular Polymerization Under Kinetic and Thermodynamic Control,” Angewandte Chemie International Edition 58 (2019): 16730–16740, 10.1002/anie.201905724. 31271244 PMC6900041

[15] Y. Xue , Y. Jin , Y. Zhang , F. Han , and F. Wang , “Reversible Control of Pathway Complexity in Supramolecular Polymerization,” Chemistry of Materials 36 (2024): 6347–6369, 10.1021/acs.chemmater.4c01265.

[16] E. J. Johnston , E. L. Rylott , E. Beynon , A. Lorenz , V. Chechik , and N. C. Bruce , “Monodehydroascorbate Reductase Mediates TNT Toxicity in Plants,” Science 349 (2015): 1072–1075, 10.1126/science.aab3472. 26339024

[17] J. Leira‐Iglesias , A. Tassoni , T. Adachi , M. Stich , and T. M. Hermans , “Oscillations, Travelling Fronts and Patterns in a Supramolecular System,” Nature Nanotechnology 13 (2018): 1021–1027, 10.1038/s41565-018-0270-4. 30323361

[18] M. G. Howlett , A. H. J. Engwerda , R. J. H. Scanes , and S. P. Fletcher , “An Autonomously Oscillating Supramolecular Self‐Replicator,” Nature Chemistry 14 (2022): 805–810, 10.1038/s41557-022-00949-6. 35618766

[19] R. K. Dubey and F. Würthner , “Lithium‐Ion‐Templated Supramolecular Polymerization of a Perylene Bisimide Dye,” Nature Chemistry 15 (2023): 1111–1119.

[20] F. Würthner , C. R. Saha‐Möller , B. Fimmel , S. Ogi , P. Leowanawat , and D. Schmidt , “Perylene Bisimide Dye Assemblies as Archetype Functional Supramolecular Materials,” Chemical Reviews 116 (2016): 962–1052, 10.1021/acs.chemrev.5b00188. 26270260

[21] C. Ji , W. Cheng , Q. Yuan , K. Müllen , and M. Yin , “From Dyestuff Chemistry to Cancer Theranostics: The Rise of Rylenecarboximides,” Accounts of Chemical Research 52 (2019): 2266–2277, 10.1021/acs.accounts.9b00221. 31373482

[22] C. Schaack , A. M. Evans , F. Ng , M. L. Steigerwald , and C. Nuckolls , “High‐Performance Organic Electronic Materials by Contorting Perylene Diimides,” Journal of the American Chemical Society 144 (2022): 42–51, 10.1021/jacs.1c11544. 34937338

[23] A. K. Dwivedi , M. Pandeeswar , and T. Govindaraju , “Assembly Modulation of PDI Derivative as a Supramolecular Fluorescence Switching Probe for Detection of Cationic Surfactant and Metal Ions in Aqueous media,” ACS Applied Materials and Interfaces 6 (2014): 21369–21379, 10.1021/am5063844. 25405529

[24] P. Yu , Y. Li , H. Zhao , et al., “1D Mixed‐Stack Cocrystals Based on Perylene Diimide Toward Ambipolar Charge Transport,” Small 17 (2021): 2006574, 10.1002/smll.202006574. 33825322

[25] E. Shirman , A. Ustinov , N. Ben‐Shitrit , et al., “Stable Aromatic Dianion in Water,” Journal of Physical Chemistry B 112 (2008): 8855–8858, 10.1021/jp8029743. 18597517

[26] S. Kumagai , H. Ishii , G. Watanabe , et al., “Nitrogen‐Containing Perylene Diimides: Molecular Design, Robust Aggregated Structures, and Advances in n‐Type Organic Semiconductors,” Accounts of Chemical Research 55 (2022): 660–672, 10.1021/acs.accounts.1c00548. 35157436

[27] E. Krieg , E. Shirman , H. Weissman , et al., “Supramolecular Gel Based on a Perylene Diimide Dye: Multiple Stimuli Responsiveness, Robustness, and Photofunction,” Journal of the American Chemical Society 131 (2009): 14365–14373, 10.1021/ja903938g. 19807182

[28] C. Chen , J. S. Valera , T. B. M. Adachi , and T. M. Hermans , “Efficient Photoredox Cycles to Control Perylenediimide Self‐Assembly,” Chemistry–A European Journal 29 (2023): e202202849, 10.1002/chem.202202849. 36112270 PMC10098730

[29] C. Chen and Z. Guan , “Precise Control of Dissipative Self‐assembly by Light and Electricity,” Chemistry–A European Journal 29 (2023): e202300347, 10.1002/chem.202300347. 36737408

[30] A. Bartnik , L. Zeinalvand , D. Kodira , P. R. Prasad , J. Patterson , and S. Sim , “Redox‐Enabled Pathway Complexity in Supramolecular Hydrogels,” Chemistry–A European Journal 31 (2025): e202500901, 10.1002/chem.202500901. 40310758

[31] C. Shao , M. Grüne , M. Stolte , and F. Würthner , “Perylene Bisimide Dimer Aggregates: Fundamental Insights Into Self‐Assembly by NMR and UV/Vis Spectroscopy,” Chemistry–A European Journal 18 (2012): 13665–13677, 10.1002/chem.201201661. 23001630

[32] A. D. Shaller , W. Wang , A. Li , et al., “Sequence‐controlled Oligomers Fold Into Nanosolenoids and Impart Unusual Optical Properties,” Chemistry–A European Journal 17 (2011): 8350–8362, 10.1002/chem.201100612. 21671296

[33] Z. Chen , A. Lohr , C. R. Saha‐Möller , and F. Würthner , “Self‐Assembled π‐stacks of Functional Dyes in Solution: Structural and Thermodynamic Features,” Chemical Society Reviews 38 (2009): 564–584, 10.1039/B809359H. 19169466

[34] C. Naranjo , A. Doncel‐Giménez , R. Gómez , J. Aragó , E. Ortí , and L. Sánchez , “Solvent‐Dependent Self‐Assembly of N‐annulated Perylene Diimides. From Dimers to Supramolecular Polymers,” Chemical Science 14 (2023): 9900–9909, 10.1039/D3SC03372D. 37736635 PMC10510848

[35] S. Ghosh , X. Q. Li , V. Stepanenko , and F. Würthner , “Control of H‐ and J‐type π Stacking by Peripheral Alkyl Chains and Self‐Sorting Phenomena in Perylene Bisimide Homo‐ and Heteroaggregates,” Chemistry – A European Journal 14 (2008): 11343–11357, 10.1002/chem.200801454. 19009569

[36] F. Würthner , C. Bauer , V. Stepanenko , and S. Yagai , “A Black Perylene Bisimide Super Gelator With an Unexpected J‐type Absorption Band,” Advanced Materials 20 (2008): 1695–1698, 10.1002/adma.200702935.

[37] D. Liu , J. Wang , X. Bai , R. Zong , and Y. Zhu , “Self‐Assembled PDINH Supramolecular System for Photocatalysis Under Visible Light,” Advanced Materials 28 (2016): 7284–7290, 10.1002/adma.201601168. 27311128

[38] S. Ogi , K. Sugiyasu , S. Manna , S. Samitsu , and M. Takeuchi , “Living Supramolecular Polymerization Realized Through a Biomimetic Approach,” Nature Chemistry 6 (2014): 188–195, 10.1038/nchem.1849. 24557132

[39] M. Wehner and F. Würthner , “Supramolecular Polymerization Through Kinetic Pathway Control and Living Chain Growth,” Nature Reviews Chemistry 4 (2020): 38–53, 10.1038/s41570-019-0153-8.

[40] M. Kumar , N. L. Ing , V. Narang , N. K. Wijerathne , A. I. Hochbaum , and R. V. Ulijn , “Amino‐acid‐encoded Biocatalytic Self‐Assembly Enables the Formation of Transient Conducting Nanostructures,” Nature Chemistry 10 (2018): 696–703, 10.1038/s41557-018-0047-2. 29713031

[41] C. Zhao , Y. Wang , Y. Jiang , et al., “Handedness‐Inverted and Stimuli‐Responsive Circularly Polarized Luminescent Nano/Micromaterials through Pathway‐Dependent Chiral Supramolecular Polymorphism,” Advanced Materials 36 (2024): 2403329, 10.1002/adma.202403329. 38625749

[42] R. N. V. Krishna Deepak and R. Sankararamakrishnan , “N–H···N Hydrogen Bonds Involving Histidine Imidazole Nitrogen Atoms: A New Structural Role for Histidine Residues in Proteins,” Biochemistry 55 (2016): 3774–3783, 10.1021/acs.biochem.6b00253. 27305350

[43] D. Görl , X. Zhang , and F. Würthner , “Molecular Assemblies of Perylene Bisimide Dyes in Water,” Angewandte Chemie International Edition 51 (2012): 6328–6348. 22573415 10.1002/anie.201108690

[44] T. Fukui , S. Kawai , S. Fujinuma , et al., “Control Over Differentiation of a Metastable Supramolecular Assembly in One and Two Dimensions,” Nature Chemistry 9 (2017): 493–499, 10.1038/nchem.2684. 28430199

[45] M. M. J. Smulders , M. M. L. Nieuwenhuizen , T. F. A. De Greef , P. Van Der Schoot , A. P. H. J. Schenning , and E. W. Meijer , “How to Distinguish Isodesmic From Cooperative Supramolecular Polymerisation,” Chemistry–A European Journal 16 (2010): 362–367, 10.1002/chem.200902415. 19921721

[46] M. Wehner , M. I. S. Röhr , M. Bühler , V. Stepanenko , W. Wagner , and F. Würthner , “Supramolecular Polymorphism in One‐Dimensional Self‐Assembly by Kinetic Pathway Control,” Journal of the American Chemical Society 141 (2019): 6092–6107, 10.1021/jacs.9b02046. 30892890

[47] W. Wagner , M. Wehner , V. Stepanenko , S. Ogi , and F. Würthner , “Living Supramolecular Polymerization of a Perylene Bisimide Dye Into Fluorescent J‐Aggregates,” Angewandte Chemie International Edition 56 (2017): 16008–16012, 10.1002/anie.201709307. 29035005

[48] S. Ogi , V. Stepanenko , K. Sugiyasu , M. Takeuchi , and F. Würthner , “Mechanism of Self‐Assembly Process and Seeded Supramolecular Polymerization of Perylene Bisimide Organogelator,” Journal of the American Chemical Society 137 (2015): 3300–3307, 10.1021/ja511952c. 25689054

[49] K. Jalani , A. D. Das , R. Sasmal , S. S. Agasti , and S. J. George , “Transient Dormant Monomer States for Supramolecular Polymers With Low Dispersity,” Nature Communications 11 (2020): 373, 10.1038/s41467-020-17799-w. PMC7415150 32770122

[50] K. Liu , A. Levy , C. Liu , and J. H. Olivier , “Tuning Structure‐Function Properties of π‐Conjugated Superstructures by Redox‐Assisted Self‐Assembly,” hemistry of Materials 30 (2018): 2143–2150, 10.1021/acs.chemmater.8b00518.

[51] J. P. Wojciechowski , A. D. Martin , and P. Thordarson , “Kinetically Controlled Lifetimes in Redox‐Responsive Transient Supramolecular Hydrogels,” Journal of the American Chemical Society 140 (2018): 2869–2874, 10.1021/jacs.7b12198. 29406709

[52] J. Gershberg , F. Fennel , T. H. Rehm , S. Lochbrunner , and F. Würthner , “Anti‐cooperative Supramolecular Polymerization: A New *K* _2_‐*K* Model Applied to the Self‐Assembly of Perylene Bisimide Dye Proceeding via Well‐Defined Hydrogen‐Bonded Dimers,” Chemical Science 7 (2016): 1729–1737, 10.1039/C5SC03759J. 28966774 PMC5582317

[53] M. M. J. Smulders , A. P. H. J. Schenning , and E. W. Meijer , “Insight Into the Mechanisms of Cooperative Self‐Assembly: The “Sergeants‐and‐Soldiers” Principle of Chiral and Achiral *C* _3_‐Symmetrical Discotic Triamides,” Journal of the American Chemical Society 130 (2008): 606–611, 10.1021/ja075987k. 18081281

[54] P. Jonkheijm , P. van der Schoot , A. P. H. J. Schenning , and E. W. Meijer , “Probing the Solvent‐Assisted Nucleation Pathway in Chemical Self‐Assembly,” Science 313 (2006): 80–83, 10.1126/science.1127884. 16825566

