# Supplementary material for: Electrochemically Triggered Supramolecular Polymerization Under Kinetic Control
Source: Angew Chem Int Ed Engl. 2026 Jun 24;65(35):e8733637. doi: 10.1002/anie.8733637 (PMC13502627; doi:10.1002/anie.8733637)
Supplement: Supplementary file 1 [file ANIE-65-e8733637-s001.docx]

**Supporting Information**

**Electrochemically Triggered Supramolecular Polymerization under Kinetic Control**

Eun Gyu Lee,^[a]^ Jeongse Yun,^[a,c]^ Hyoung Wook Kang, ^[a]^ Daeun Jeong,^[a]^ Seung-Ryong Kwon,* ^[a,c]^ Jong Hwa Jung,*^[a,b]^ and Sung Ho Jung*^[a,b]^

[a] Department of Chemistry, Gyeongsang National University, Jinju 52828, Republic of Korea

E-mail: srkwon@gnu.ac.kr, jonghwa@gnu.ac.kr, shjung@gnu.ac.kr

[b] Research Institute of Advanced Chemistry, Gyeongsang National University, Jinju 52828, Republic of Korea Department

[c] Research Institute of Molecular Alchemy, Gyeongsang National University, Jinju 52828, Republic of Korea Department

**1. General**

The ^1^H NMR spectra were taken on a Bruker DRX 600 and mass spectroscopy samples were analyzed on a 4800 MALDI-Tof/Tof Mass Spectrometer (SCIEX). A UV-visible spectrophotometer (Jasco V-730) was used to obtain the absorption spectra. IR spectra were obtained for CaF_2_ drilled window (Spectra-Tech) in the range 400–4000 cm^-1^, with a Jasco FT-IR 4x spectrometer. The fluorescence spectra were obtained using a spectrophotometer (Jasco FP-8250, CPL-300). Powder X-ray pattern (PXRD) was recorded on a D2 bruker model D2 PHASER X-ray diffractometer with a Cu Kα radiation source. The solvents for chemical and electrochemical experiments were DMSO (spectroscopic grade) and ultrapure Millipore MilliQ water (specific resistance, 18.2 MΩ cm). The pH values were adjusted either with a freshly prepared Tris-HCl buffer.

1.1 **Atomic Force Microscopy (AFM) studies**

Atomic force microscope (AFM) imaging was performed by using NX7 and a PPP-NCHR cantilever (Park Systems). The AFM samples were prepared by spin-coating (1000 - 3000 rpm) onto freshly cleaved HOPG, and images were recorded with the AFM operating in a Tapping mode in the air at RT with a resolution of 1024 × 1024 pixels, using moderate scan rates (0.4 Hz).

1.2 **Fourier Transform Infrared Spectroscopy**

FT-IR spectroscopic measurements were carried out using a JASCO FT/IR-4X spectrometer. Solution-phase spectra were obtained with a Falcon Mid-IR transmission accessory equipped with a temperature control module and liquid recirculator. Samples were measured in a fixed-path liquid cell fitted with CaF₂ windows and a 1 mm path length (PIKE Technologies). Solid-state FT-IR spectra were recorded on the same instrument using an ATR Pro ONE accessory (JASCO).

1.3 **Dynamic Light Scattering (DLS) Measurements**

DLS measurements were carried out using a Zetasizer Nano ZS employing a 633 nm laser at a back scattering angle of 173º.

1.4 **Preparation of aggregates (Agg-I and Agg-II) and redox experiment**

A stock solution of PDI-His was prepared by dissolving the compound in a DMSO/H₂O (1:1, v/v) mixture to a final volume of 3 mL. Preparation of Agg-I: The solution was heated to 373 K for 5 min and then rapidly cooled to 298 K at a cooling rate of 10 K·min⁻¹. Preparation of Agg-II: The Agg-I solution was stirred at 298 K and 400 rpm for 60 h to induce transformation to Agg-II. The resulting samples were subsequently characterized by UV-vis absorption.

For chemical reduction, a freshly prepared aqueous solution of sodium dithionite (SDT, Na₂S₂O₄) was used as the reducing agent. A defined volume of the SDT solution (1.0-10 eq. relative to **PDI-His**, final dilution: 1%) was added to the aggregate dispersion at 298 K under gentle mixing. The reduction process was carried out under ambient atmosphere, the redox process was monitored by time-dependent UV-vis absorption spectroscopy until no further changes were observed.

1.5 **Computational calculations**

Density functional theory (DFT) was employed to calculate the molecular structures. First, the conformers with the lowest energy were identified at the AM1 level for conformational searches. These conformers were further optimized at the B3LYP/6-31+G(d) level using Spartan 24 software to investigate the lowest-energy conformer.

1.6 **Electrochemical measurement**

All electrochemical experiments were conducted using a potentiostat (CHI760E, CH Instruments, US). For cyclic voltammetry, a glassy carbon working electrode (ALS, Japan), a leak-free reference electrode (Innovative Instruments, USA), and a platinum wire (Merck, US) were used to construct a three-electrode electrochemical cell. The electrolytes—LiCl (Merck, US) for the PDI monomer solution in pure DMSO and KCl (Merck, US) for Agg-2 dispersed in a 1:1 mixture of DMSO and water were added to the PDI solutions by mixing with concentrated aqueous stocks at a 100:1 volumetric ratio to achieve the target concentration. The glassy carbon working electrodes were gently polished with colloidal alumina solutions (1.0, 0.3 and 0.05 μm in diameter) to expose a fresh surface. Before conducting cyclic voltammetry, 2 mL of the prepared PDI solution was transferred to a homemade Teflon vessel, ensuring that all electrodes were fully immersed in the solution. To suppress oxygen reduction reaction, nitrogen gas (daepoong, Korea, 99.999%) was gently bubbled through the solution via a silicone tube for 10 minutes. During the measurement, nitrogen gas was continuously supplied to the headspace of the Teflon vessel to prevent oxygen dissolution. The potential of the leak-free reference electrode was checked against commercial Ag/AgCl (3 M KCl) aqueous reference electrode (BASi, US), and the potential in the cyclic voltammogram was displayed against the potential of Ag/AgCl (3 M KCl).

For electrochemical conductivity measurements, interdigitated gold electrode with 50 μm spacing was employed (Platypus Technologies, US). After constructing the electrochemical setup by mounting the electrode in the homemade Teflon vessel and loading the sample solution, sine-wave alternating voltage of 5 mV amplitude and the frequency varied from 10 kHz to 1 MHz was applied. The interfacial resistance was obtained by fitting the impedance response to a Randles circuit, and its reciprocal was normalized by the geometric area of one side of the interdigitated electrode to yield the area-normalized conductance. The conductivity measurements were conducted repeatedly for 72 hours after preparing a fresh 100 μM PDI solution in the 1:1 mixture of DMSO and H_2_O.

For heterogeneous electrode stimulus-responsive supramolecular polymerization, a potential of +1 V was applied for 1 hour in 2 mL of 100 μM Agg-I solution in the three-electrode electrochemical cell. After the potential application, the HOPG electrode was gently washed with the 1:1 mixture of DMSO and H_2_O.

1.7 **Determination of Degree of Aggregation**

The degree of aggregation (α) was determined using both temperature- and concentration-dependent spectroscopic methods.

For the temperature-dependent UV-Vis measurements, the degree of aggregation was defined as

$$\alpha(T) =\frac{\varepsilon(T)-\varepsilon_{M}}{\varepsilon_{A}-\varepsilon_{M}}$$

where ε(*T*) is the apparent extinction coefficient at a given temperature, determined from UV–Vis absorption measurements. ε*_M_* and ε*_A_* represent the extinction coefficients of the fully molecularly dissolved monomeric state and the fully aggregated state, respectively. These values were obtained by extrapolating the experimentally measured extinction coefficients at the high- and low-temperature limits.

For Agg-I, the temperature-dependent aggregation behavior was monitored during the cooling process. In contrast, for Agg-II, the sample was allowed to completely transform from Agg-I into Agg-II at room temperature, and the subsequent heating process was monitored.

**2. Synthesis and Characterization**

Unless otherwise noted, chemical reagents and solvents were purchased from commercial suppliers (Tokyo Chemical Industry (TCI), Sigma-Aldrich (Merck)) and used without further purification.

**2.1 Material synthesis**

All chemical reagents were purchased commercially without further purification unless otherwise noted. 0.76

**Synthesis of compound PDI-His.** 3,4,9,10-perylenetetracarboxylic dianhydride (PDI) (0.3 g, 0.77 mol), Glycyl-*L*-histidine (0.4 g, 1.6 mmol), and imidazole (2.08 g) were added into a reaction and receiver tube with two standard taper flask followed by heating at 120 ^o^C for 1 day, vigorous stirring, and a nitrogen atmosphere. The reaction mixture was allowed to cool to 90 ^o^C, transferred into distilled water and filtered. The filtrate was acidified with 2.0N HCl and stirred overnight. The precipitate was filtered, washed with an excess of distilled water, acetone, and dried under vacuum at 50 ^o^C to obtain the product. Yield: 51.6 %. ^1^H NMR(600MHz, *DMSO*-*d*_6_) *δ*_H_ 8.96 (4H, d), 8.61 (4H, d), 8.43 (2H, br), 8.27 (2H, br), 7.37 (2H, s), 7.13 (2H, s), 5.68 (2H, br), 4.75 (2H, br), 4.58 (2H, br), 2.08 (2H, s). ^13^C NMR(150MHz, *DMSO*-*d*_6_) *δ*_C_ 172.4., 170.9, 166.32, 135.1, 134.9, 130.2, 127.5, 123.8, 121.2, 117.3, 116.2, 53.0,44.8, 29.1. Elemental Analysis: Found C, 61.24; H, 3.18; N, 14.18; calcd C, 61.54; H, 3.62; N, 14.35; O, 20.49 for C_40_H_28_N_8_O_10_. MALDI-TOF mass: m/z Found 781.193 [M+H]^+^; calcd. 780.710 for C_40_H_28_N_8_O_10_.

**3. Scheme, Figures, and Tables**

**Scheme S1.** Synthesis of method **PDI-His**.

**Figure S1**. (A) UV-Vis absorption spectra of **PDI-His** (100 μM) at different water fractions in a mixture of DMSO and H_2_O. (B) Plot of absorbance at 527 nm and (c) A_0-1_/A_0-0_ ratios of **PDI-His** as a function of water fraction.

**Figure S2**. CD Spectra of monomeric **PDI-His** in DMSO (black solid line), Agg-I (red solid line), and Agg-II (green solid line) in a mixture of DMSO/H_2_O (1:1, v/v); concentration of **PDI-His** =100 μM.

**Figure S3.** DFT-optimized structures of trimeric **PDI-His** calculated at the B3LYP/6-31G(d) level; (A) side and (B) top view.

**Figure S4.** P-XRD of Agg-I and Agg-II.

**Figure S5.** FT-IR spectra of the (A) N-H stretching vibration and (B) C=O and C-N stretching vibration for the solution of monomeric **PDI-His** in DMSO (black line), Agg-I (red line), and Agg-II (blue line) in a mixture of DMSO/H_2_O (1:1, v/v).

**Figure S6**. DFT-optimized structure of (A) monomeric and (B) dimeric **PDI-His** calculated at the B3LYP/6-31G(d) level.

**Figure S7.** (A-E) Temperature-dependent UV-vis spectra of **PDI-His** (Agg-I) at different concentrations (50–200 μM) from 373 to 283 K at a cooling rate of 1 K min^−1^ in a mixture of DMSO/H_2_O (1:1, v/v).

**Figure S8.** (A) Degree of aggregation (α_dimer_, monitored at 527 nm) of **PDI-His** as a function of concentration in DMSO/H₂O (1:1, v/v), obtained by fitting the absorption data to a dimerization model (Eq. S1).^[52]^ Here, ε_mono_ and ε_dimer_ denote the molar absorption coefficients of the monomer and dimer, respectively, and ε(c) represents the apparent absorption coefficient at a given concentration. (B) Van’t Hoff plot of ln *K*_d_ versus *T*^-^¹, showing a linear relationship. The standard enthalpy (Δ*H*°) and entropy (Δ*S*°) changes were determined from Eq. S2 (Correlation coefficient, R² = 0.995).

(S1)

(S2)

**Table S1**. Aggregation constants (*K*_d_) for Agg-I formation in a mixture of DMSO/H_2_O (1:1, v/v) at different temperatures.

| *T* (K) | *K*_d_ (M^-1^) |
| --- | --- |
| 330 | 7.8 x 10^2^ ± 30 |
| 325 | 1.3 x 10^3^ ± 59 |
| 320 | 2.2 x 10^3^ ± 109 |
| 315 | 3.7 x 10^3^ ± 185 |
| 310 | 6.5 x 10^3^ ± 325 |
| 305 | 1.2 x 10^4^ ± 407 |
| 300 | 2.4 x 10^4^ ± 622 |
| 295 | 5.9 x 10^4^ ± 1331 |
| 290 | 2.0 x 10^5^ ± 2599 |

**Figure S9*.*** (A-E) Temperature-dependent UV-vis spectra of the **PDI-His** (Agg-II) at different concentrations (50–200 μM) were observed from 283 K to 373 K at a heating rate of 1 K min^−1^ in a mixture of DMSO/H_2_O (1:1, v/v).

**Figure S10*.*** (A) Temperature-dependent degree of Agg-II calculated from the apparent absorption coefficients at 600 nm at different total concentrations (*c*_T_) in a mixture of DMSO/H_2_O (1:1, v/v) during the heating process. The curves show the resulting fits to the cooperative model proposed by Meijer and co-workers (Eq. S3).^[53,54]^ (B) Natural logarithm of the reciprocal *c*_T_ as a function of the reciprocal *T*_e_ observed upon heating a solution of Agg-II at a rate of 1 K min^–1^ (Correlation coefficient, R² = 0.996). The standard enthalpy change (Δ*H*°) and entropy change (Δ*S*°) values are determined from Eq. S4.

(S3)

(S4)

**Table S2**. Thermodynamic parameters for Agg-II obtained by fitting the temperature-dependent degree of aggregation in DMSO/H_2_O (1:1, v/v) to the cooperative model.

| *c*_T_ (μM) | *a*_SAT_ | *T*_e_ (K) | *ΔH_e_* (kJ mol^-1^) |
| --- | --- | --- | --- |
| 200 | 1.013 | 349.6 ± 0.013 | -100.3 ± 0.52 |
| 150 | 1.010 | 347.9 ± 0.012 | -100.3 ± 0.52 |
| 100 | 1.005 | 344.1 ± 0.012 | -102.8 ± 0.55 |
| 75 | 1.008 | 341.4 ± 0.013 | -100.8 ± 0.56 |
| 50 | 1.012 | 388.1 ± 0.012 | -100.5 ± 0.56 |

**Figure S11*.*** (A-D) Time-dependent UV-vis spectra of Agg-I upon addition of SDT (1.0-4.0 eq.) in a mixture of DMSO/H_2_O (1:1, v/v) at 293 K.

Figure S12. (A) Time-dependent UV-vis spectra of Agg-II in a mixture of DMSO/H₂O (1:1, v/v) at 293 K after the addition of SDT (5.0 eq.). (B) Plot of absorption of Agg-II at 546 nm after addition of SDT. (C) AFM image of Agg-II after addition of SDT.

**Figure S13.** (A) Photographs showing the color changes corresponding to formation of PDI^2-^ (pink) and PDI^•-^ (violet) upon the addition of SDT (40 eq.) to Agg-I in a mixture of DMSO/H_2_O (1:1, v/v) at 293 K. (B) Time-dependent UV-vis spectra showing the spectral evolution and corresponding color changes assigned to PDI^2-^ (pink) and PDI^•-^ (violet) in transformation from Agg-I into Agg-II.

Figure S14. (A) Time-dependent UV–Vis absorption spectral changes of PDI-His after the addition of SDT (5.0 eq.) to Agg-I in a mixture of DMSO/H₂O (1:1, v/v) at 293 K under deoxygenated conditions using a sealed cuvette without air exposure. (B) UV–Vis absorption spectrum of the reduced PDI species (PDI-His^2−^) obtained after 150 min, showing suppression of the transformation from Agg-I to Agg-II under deoxygenated conditions.

Figure S15. DFT-optimized structures of dianionic PDI–His (PDI-His^2-^) calculated at the B3LYP/6-31+G(d) level.

**Figure S16.** AFM images of Agg-II_seed_ obtained after chemical redox reaction (5.0 eq, SDT) in a mixture of DMSO/H_2_O (1:1 v/v). The original AFM image corresponds to the same sample shown in Figure 4c in the main text. Fiber lengths were analyzed only for supramolecular fibers with both termini clearly visible in the image, as indicated by the yellow lines.

**Figure S17.** Dynamic light scattering (DLS) spectra show that the hydrodynamic radius decreases as the equivalent of the reduction agent (SDT) increases.

Note: Since DLS will treat supramolecular polymers as spherical aggregates, the obtained values will not be accurate and can only be used to show the trend.

**Figure S18.** AFM images of supramolecular polymers obtained after chemical redox reaction in a mixture of DMSO/H_2_O (1:1 v/v) at different SDT concentrations: (A) 4.0 eq., (B) 3.0 eq., (C) 2.0 eq., (D) 1.0 eq. (E-G) Corresponding length distribution analyses were derived from supramolecular polymers formed under chemical redox conditions (approximately 100 individual supramolecular polymers were traced for each condition).

*Note:* At 1.0 equivalent of SDT, the length of supramolecular polymers exceeded the measurable range, preventing reliable length analysis.

**Figure S19**. AFM images obtained from seeded living supramolecular polymerization in a mixture of DMSO and H_2_O (5:5 v/v) under conditions: ratio of [Agg-II_Seed_]:[Agg(I)] = (A) Seed, (B) 1:1, (C) 1:2, (D) 1:3 and (E) 1:4. (F-J) Corresponding length distribution histograms were obtained from seeded living supramolecular polymerization (approximately 100 individual supramolecular polymers were traced for each condition).

**Figure S20**. Cyclic voltammogram of monomeric **PDI-His** and Agg-II. (Scan rate = 10 mV/s)

**Figure S21**. CD Spectra of Agg-II prepared by the electrochemical method (block solid line) and time-evolution (red solid line).

**Figure S22**. FT-IR Spectra of (A) N-H stretching vibration and (B) C=O and C-N stretching vibration for the solution of Agg-II prepared by the electrochemical method (red solid line) and time-evolution (blue solid line).

**Figure S23**. (A) AFM image and corresponding (B) length distribution of Agg-II_seed_ obtained after electrochemical stimuli to Agg-I.

**Figure S24.** ^1^H NMR spectrum (600 MHz) of **PDI-His** in *DMSO*-*d*_6_.

**Figure S25.** ^13^C NMR spectrum (150 MHz) of **PDI-His** in *DMSO*-*d*_6_.

**Figure S26.** MALDI-TOF Mass Spectrum of **PDI-His**.
